# Complete Genome Sequence of an Influenza D Virus Strain Identified in a Pig with Subclinical Infection in the United States

**DOI:** 10.1128/MRA.01462-18

**Published:** 2019-01-24

**Authors:** Peter Thielen, Jacqueline M. Nolting, Sarah W. Nelson, Thomas S. Mehoke, Craig Howser, Andrew S. Bowman

**Affiliations:** a The Johns Hopkins Applied Physics Laboratory, Laurel, Maryland, USA; b Department of Veterinary Preventive Medicine, The Ohio State University, Columbus, Ohio, USA; Queens College

## Abstract

Influenza D virus was first described in 2011 from a pig with respiratory disease; however, recent evidence indicates that cattle are the major viral reservoir. Here, we describe the genome sequence of the eighth complete swine-origin influenza D virus deposited into GenBank, D/swine/Kentucky/17TOSU1262/2017, which was collected at a 2017 swine exhibition.

## ANNOUNCEMENT

Influenza D virus (IDV) was first isolated from ill swine in the United States in 2011, and it shares approximately 50% similarity to influenza C virus (1). Subsequently, IDV has been detected in several livestock hosts; serological surveillance indicates widespread circulation in cattle across North America, Asia, and Europe (2–7), making IDV a previously unappreciated animal pathogen with cattle as a primary reservoir (8).

During active influenza A virus (IAV) surveillance, we collected nasal swabs from 200 pigs in a population of 933 exhibition swine at a 2017 show in Kentucky. Sample collection was approved by the Institutional Animal Care and Use Committee at The Ohio State University. During sample collection, we observed each animal for influenza-like illness (i.e., cough and nasal discharge). We screened all samples for IAV using the Mag-Bind viral DNA/RNA 96 kit (Omega Bio-tek, Inc., Norcross, GA, USA) and the VetMAX-Gold SIV real-time reverse transcription-PCR (rRT-PCR) detection kit (Life Technologies Corp., Austin, TX, USA). IAV was molecularly detected in 85 samples, which subsequently yielded 9 IAV isolates through virus isolation (9). One rRT-PCR-positive but IAV-isolation-negative sample produced cytopathic effects consistent with IAV and agglutinated turkey red blood cells similar to IAV but was IAV antigen negative (Flu DETECT swine; Zoetis, Parsippany, NJ, USA). The isolate was IAV negative using rRT-PCR, but it was positive for IDV (1). No clinical signs of disease were noted in the pig from which this IDV isolate was recovered.

Viral RNA was extracted as described above. Illumina RNA sequencing libraries were generated using NEBNext Ultra RNA library preparation reagents for Illumina (New England Biolabs, Ipswich, MA, USA), with a protocol modification eliminating oligo(dT) selection prior to random-primed cDNA synthesis and subsequent library construction. Indexed libraries were pooled and sequenced on a NextSeq 500 instrument, with 150-bp paired-end v2 sequencing chemistry (Illumina, San Diego, CA, USA), resulting in 25 million read pairs for the sample. Data were quality filtered to retain Q30 or greater reads prior to alignment to the D/bovine/Minnesota/729/2013 reference sequence using Bowtie 2 (version 2.1.0) with the “–very-sensitive-local” option, converted to sorted BAM files using SAMtools, with the consensus sequence generated with SAMtools mpileup. Overall, 53,273 read pairs aligned to the reference. Iterative reference alignment was used to eliminate mismatches and indels. The total sequence length is 12,264 nucleotides, with a G+C content of 41.5%.

Currently, GenBank contains sequence data for 74 IDVs; 57, 16, and 1 of these viruses originated from bovine, swine, and caprine hosts, respectively. Complete genome sequences are available for only 23 of the IDVs, with 16 of these originating from cattle and 7 from swine. With >98% identities, the closest BLAST matches for all seven D/swine/Kentucky/17TOSU1262/2017 genome segments were from bovine hosts. While the lack of publicly available IDV sequences hindered investigation, the IDV in this healthy pig likely resulted through spillover from cattle. This observation supports the need for additional monitoring of IDV to identify genomic diversity and transmissibility between species.

### Data availability.

The consecutive GenBank accession numbers MK054178 to MK054184 contain the genome sequence of D/swine/Kentucky/17TOSU1262/2017. The accession number SRX5106712 in the Sequence Read Archive holds the Illumina data.
